# Sexualizing Media Use and Self-Objectification: A Meta-Analysis

**DOI:** 10.1177/0361684317743019

**Published:** 2017-12-15

**Authors:** Kathrin Karsay, Johannes Knoll, Jörg Matthes

**Affiliations:** 1 Department of Communication, University of Vienna, Vienna, Austria

**Keywords:** meta-analysis, self-objectification, body image, media use

## Abstract

Objectification theorists suggest that exposure to sexualizing media increases self-objectification among individuals. Correlational and experimental research examining this relation has received growing attention. The aim of this meta-analysis was to investigate the influence of sexualizing media use on self-objectification among women and men. For this purpose, we analyzed 54 papers yielding 50 independent studies and 261 effect sizes. The data revealed a positive, moderate effect of sexualizing media on self-objectification (*r* = .19). The effect was significant and robust, 95% CI [.15, .23], *p* < .0001. We identified a conditional effect of media type, suggesting that the use of video games and/or online media led to stronger self-objectification effects when compared to television use. Other sample characteristics or study characteristics did not moderate the overall effect. Thus, our findings highlight the importance of sexualizing media exposure on women’s and men’s objectified self-concept. We discuss future research directions and implications for practice. We hope that the article will stimulate researchers in their future work to address the research gaps outlined here. Moreover, we hope that the findings will encourage practitioners and parents to reflect on the role of the use of sexualizing media in the development of individuals’ self-objectification. *Additional online materials for this article are available on PWQ’s website at http://journals.sagepub.com/doi/suppl10.1177/0361684317743019*

Today’s mainstream media (e.g., television, print materials, video games, social networking sites) are marked by an emphasis on sexual appearance, physical beauty, and sexual appeal to others (American Psychological Association [APA], 2007). This type of presentation is labeled sexualization (Fredrickson & Roberts, 1997; Ward, 2016; Zurbriggen, 2013). Sexualizing media content has been criticized for many reasons. For instance, exposure to sexualizing media has been related to reinforced gender stereotypes (e.g., Galdi, Maass, & Cadinu, 2014), an increased acceptance of rape myths (e.g., Fox, Ralston, Cooper, & Jones, 2015), and increased body dissatisfaction (e.g., Halliwell, Malson, & Tischner, 2011). In the remainder of this article, we use the term “sexualized” when we refer to the presentation of individuals and characters in media. We speak of “sexualizing” content when referring to the effects of the media individuals and characters on the viewer.

Drawing on objectification theory (Fredrickson & Roberts, 1997), our main goal in the current study was to explore the extent, and under which conditions, sexualizing media elicit self-objectification among individuals. Objectification theorists posit that the experience and observation of sexual objectification acculturates women and men to internalize an objectified view of the self. This view involves adopting a third-person perspective of the body and is manifested by chronic attention to one’s own physical appearance, which is defined as self-objectification (Fredrickson & Roberts, 1997; McKinley & Hyde, 1996).

Many researchers have empirically investigated the relation of sexualizing media use and self-objectification (e.g., Andrew, Tiggemann, & Clark, 2016; Aubrey, 2006a; de Vries & Peter, 2013; Grabe & Hyde, 2009; Grey, Horgan, Long, Herzog, & Lindemulder, 2016; Karsay & Matthes, 2015; Manago, Ward, Lemm, Reed, & Seabrook, 2015; Vandenbosch & Eggermont, 2012). However, the growing literature, including cross-sectional surveys, panel surveys, and experimental research, has yielded mixed results. Consequently, scholars have not yet arrived at a consensus or a conclusive judgment about the role of sexualizing media use in the development of self-objectification. We aimed our meta-analytic research at addressing this need.

## Objectification Theory

Objectification theory (Fredrickson & Roberts, 1997) and discussions about objectified body consciousness (McKinley & Hyde, 1996) have applied feminist principles in order to explain women’s experiences of sexualization and its negative consequences on women’s well-being. Theorists posit that from an early age on, women’s bodies are looked at, commented on, and evaluated by others. Girls and women learn from experienced and observed sexual objectification that (sexual) attractiveness is a central aspect of the feminine gender role, and therefore a goal for which they must strive (Fredrickson & Roberts, 1997). Objectification theory has continuously been expanded to more diverse populations, including men, sexual minorities, and ethnic minorities (Fredrickson, Hendler, Nilsen, & O’Barr, 2011).

Sexual objectification is defined as the practice of viewing, using, and/or valuing a person as an object (i.e., a thing) whose worth is based primarily for his or her physical and sexual attractiveness (Fredrickson & Roberts, 1997). Sexually objectifying experiences are not exclusively sexual in nature but also include the societal pressure to create, present, maintain, and always improve an attractive appearance (i.e., the thin-ideal for women; the muscular-ideal for men; Moradi, 2010, 2011; Zurbriggen, 2013). Thus, sexual objectification may occur in many ways and ranges from depictions of an ideal body type, to (unwanted) evaluations of one’s own body (e.g., stares, whistles, sexual comments), or sexual harassment (Kozee, Tylka, Augustus-Horvath, & Denchik, 2007; Moradi, 2011).

Fredrickson and Roberts (1997) treated sexual objectification and sexualization as interchangeable terms. In accord with the Task Force on the Sexualization of Girls, we prefer the term sexualization because it includes sexual objectification (APA, 2007). According to the APA, sexualization occurs whenever (a) a person’s value is determined primarily or only from their sexual appeal or behavior, to the exclusion of other characteristics; (b) a person is held to a standard that equates narrowly defined physical attractiveness with being sexy; (c) a person is sexually objectified; or (d) sexuality is inappropriately imposed upon a person. Any of these conditions serve as an indicator for sexualization.

Media play a crucial role in exposure to sexualizing images, text, sounds, and experiences (Fredrickson & Roberts, 1997). Results from numerous content analyses have shown that sexualization is omnipresent in a wide range of media types, like music television (Aubrey & Frisby, 2011; Vandenbosch, Vervloessem, & Eggermont, 2013), print magazines (Stankiewicz & Rosselli, 2008), video games (Burgess, Stermer, & Burgess, 2007), and social networking sites (Hall, West, & McIntyre, 2012; Kapidzic & Herring, 2015).

## Self-Objectification

Moradi (2011) has theorized that sexualizing experiences lead to an internalization of both the paramount importance of how one “appears” and beauty ideals which, in turn, leads to self-objectification. According to objectification theory (Fredrickson & Roberts, 1997), self-objectification accounts for the psychological mechanism that translates experiences of sexualization at the cultural level to psychological and behavioral features of mental health and well-being at the individual level (Calogero, Tantleff-Dunn, & Thompson, 2011; Moradi, 2010, 2011; Moradi & Huang, 2008). For example, empirical studies have shown that self-objectification predicted greater body shame and greater appearance anxiety (Moradi & Huang, 2008).

The construct of self-objectification is conceptualized as a learned *trait* (Calogero, 2011). However, it can also be elicited momentarily, such as through media use, and can lead to a *state* of self-objectification (Calogero, 2011, Moradi & Huang, 2008). There have been different approaches to operationalizing self-reported *trait self-objectification* because researchers understand it as a multifaceted concept (Calogero, 2011; Fredrickson & Roberts, 1997; Vandenbosch & Eggermont, 2012, 2013). Self-objectification comprises cognitive components, such as valuing appearance over competence (as measured by the Self-Objectification Questionnaire [SOQ]; Noll & Fredrickson, 1998), and behavioral components, such as engaging in chronic body monitoring (as measured by the Surveillance subscale of the Objectified Body Consciousness Scale [OBCS]; McKinley & Hyde, 1996). The SOQ and the OBCS subscale have shown low to moderate intercorrelations with each other (e.g., Aubrey, 2006a; Calogero, Herbozo, & Thompson, 2009; Vandenbosch & Eggermont, 2015a). Body surveillance, however, has been more consistently linked to negative outcomes, such as negative body image and mental health problems, compared to self-objectification (Moradi & Huang, 2008). Although both the SOQ and the OBCS have acceptable levels of reliability and validity in a variety of samples, and these two conceptualizations of self-objectification do overlap, they are not equivalent (Calogero, 2011; Moradi & Huang, 2008).

Typically, in experimental research, design-induced *state self-objectification* has been measured by applying Fredrickson, Roberts, Noll, Quinn, and Twenge’s (1998) Twenty Statements Test (TST). After the experimental manipulation, respondents complete up to 20 sentences beginning with “I am.” Afterward, the appearance-related statements are coded and defined as state self-objectification. Although the TST has been a commonly used measure in experimental research, it has been problematic due to low levels of variance (e.g., Aubrey, 2010; Aubrey, Henson, Hopper, & Smith, 2009; Karsay & Matthes, 2016). Researchers have also employed modified versions of the SOQ or the OBCS subscale in experimental research in order to measure states of heightened self-objectification (Calogero, 2011). As noted previously, studies on the relation between sexualizing media and self-objectification have yielded mixed results. In the following sections, we outline the current findings on the relation between sexualizing media use and self-objectification from correlational (cross-sectional and longitudinal) and experimental research. Unless noted otherwise, we use the term self-objectification if any of the above-mentioned measures was applied.

## Correlational Research

Most cross-sectional correlational studies have shown that the use of sexualizing television programs and magazines and the use of social networking sites, like Facebook or Pinterest, are positively related to self-objectification among women and men, as well as among girls and boys (Aubrey, 2007; Fardouly, Diedrichs, Vartanian, & Halliwell, 2015; Fox & Rooney, 2015; Kim, Seo, & Baek, 2015; Manago et al., 2015; Nowatzki & Morry, 2009; Tiggemann & Slater, 2014, 2015; Vandenbosch & Eggermont, 2015a). However, there are exceptions. For example, in a study by Morry and Staska (2001), neither the use of beauty nor fitness magazines was related to self-objectification among men. Mixed results were also found for the use of music television and music videos; Fardouly, Diedrichs, Vartanian, and Halliwell (2015) found no relation with self-objectification and music videos among women, but other researchers (Grabe & Hyde, 2009; Vandenbosch & Eggermont, 2015a) did for both girls and boys. Meier and Gray (2014) showed that only appearance-related, but not general, Facebook use was positively correlated with self-objectification among girls.

Only a few researchers have applied a panel (i.e., longitudinal) survey design. Aubrey (2006a) found that exposure to sexualizing television predicted trait self-objectification for both college women and men, but media exposure predicted body surveillance only for men. Doornwaard et al. (2014) also identified gender differences among adolescents. The use of sexually explicit Internet material predicted only boys’ body surveillance. In contrast, the use of social networking sites predicted body surveillance only among girls. Vandenbosch and Eggermont (2015a) identified differences between media types but not between girls and boys. The use of sexualizing mass media (e.g., magazines and music television) predicted self-objectification via the internalization of appearance ideals. However, the use of social networking sites did not predict self-objectification among adolescents. The media measure might be a possible explanation for why the findings from correlational studies varied so much. Whereas some research included a rough, undifferentiated measure of media use, others examined subsets of specific media types or media content.

In comparison to experimental research, an advantage of survey data is that participants are not forced to watch or read sexualizing media content, but rather report their habitual media exposure. However, the lack of valid and reliable measures of media exposure represents a substantial challenge in media effects research that can lead to small or inconsistent results (de Vreese & Neijens, 2016; Valkenburg & Peter, 2013). Self-reported data can be biased due to cognitive (e.g., incorrect memory) or motivational reasons (e.g., social desirability; Valkenburg & Peter, 2013).

## Experimental Research

Experimental research can lead to causal conclusions about the effects of media exposure on state self-objectification due to controlled research settings and the isolated manipulation of the independent variable. On the downside, in addition to the ethical challenges of exposing participants to sexualization content, a laboratory setting always involves an artificial environment for media use. Furthermore, the exposure to sexualizing depictions in an experimental study represents only a fraction of most participants’ actual exposure in their daily lives.

Many experimental studies have identified increased self-objectification among women after a relatively short exposure to sexualizing media content. Exposure to images of sexualized women (Aubrey et al., 2009; de Vries & Peter, 2013; Grey et al., 2016; Hopper & Aubrey, 2016), sexualizing music videos (Aubrey & Gerding, 2015; Karsay & Matthes, 2015), and sexualized video game avatars (Fox, Bailenson, & Tricase, 2013; Fox et al., 2015) increased self-objectification among young women. The few experimental studies that have investigated men showed that exposing men to sexualized images of men did not increase self-objectification (Kalodner, 1997; Michaels, Parent, & Moradi, 2013).

The few experimental studies that have been conducted with adolescents have led to divergent results. M. A. Miller (2007) found no effects after exposing girls to sexualizing images, but Daniels (2009) demonstrated an interaction effect of age and experimental condition, indicating that girls were more susceptible to the negative effects of sexualizing images, in comparison to women. We identified only one experimental study with both adolescent boys and girls as participants. Vandenbosch, Driesmans, Trekels, and Eggermont (2015) showed that playing a video game with a sexualized avatar fostered increased self-objectification among adolescents. This effect was independent of the adolescents’ gender.

## The Present Study

Meta-analysis can shed light on divergent results by calculating an overall effect size (O’Keefe, 2017). In addition, the meaning of mixed results can be clarified by adding potential moderators to the analysis. Although a number of meta-analytic studies of media use and body image exist (e.g., Barlett, Vowels, & Saucier, 2008; Grabe, Ward, & Hyde, 2008; Groesz, Levine, & Murnen, 2002; Hausenblas et al., 2013; Holmstrom, 2004; Want, 2009), there is no quantitative meta-analysis that explicitly investigates the influence of sexualizing media use on self-objectification. To date, only one quantitative meta-analysis (Grabe et al., 2008) and two narrative analyses (López-Guimerà, Levine, Sánchez-carracedo, & Fauquet, 2010; Ward, 2016) have introduced self-objectification—mainly as a subcategory of body dissatisfaction—to the analysis. We sought to contribute to the literature as follows: First, this is the first meta-analysis that explicitly investigated the hypothesis that the use of sexualizing media would increase self-objectification. Ward (2016) called for meta-analytic research that examined this relation. Second, we included the entire range of study designs in our analysis, testing possible differences between them—cross-sectional, panel, and experimental studies. Third, we included all available studies—regardless of their geographical origin—in the analyses, provided they were available in English. Hence, we did not restrict our sample to English-speaking countries, as has been the case in other meta-analyses (e.g., Grabe et al., 2008). Fourth, we used a sophisticated methodological approach. We calculated a multilevel model to take all possible effect sizes into account without aggregation and loss of information (Cheung, 2014; Field, 2015). This methodological approach allowed us to test the average effect and the roles of several theoretically relevant moderators. Finally, we have identified relevant research gaps through the current meta-analysis. Based on our findings, we proposed an agenda for future research to stimulate the fields of media effects and body image research.^1^

## Method

### Literature Search

**Figure 1.**
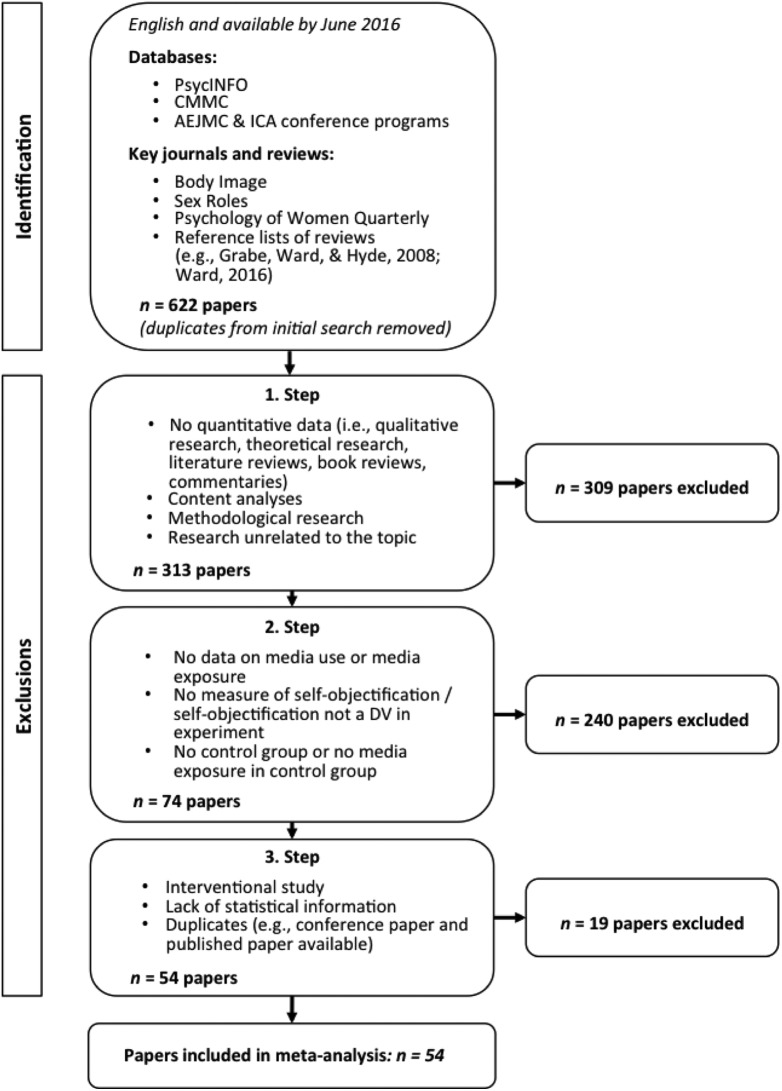
Literature search strategy for papers included in the meta-analysis.

Figure 1 illustrates our search strategy and the process of excluding papers. We collected the papers for the current study from two major databases in the fields of psychology (PsycINFO) and communication (Communication and Mass Media Complete). In addition, we browsed the programs of the annual conferences of the Association for Education in Journalism and Mass Communication and the International Communication Association. We restricted our search to research written in English and available through June 2016. We examined the databases by using the term objectification* without and in combination with media* in any available search field. Also, we used the terms body surveillance, self-surveillance, objectifi*, and objectify* in combination with the term media*, respectively. The asterisk allowed the terms to have all possible endings. To identify additional literature, we browsed through three journals (i.e., *Body Image*, *Sex Roles*, and *Psychology of Women Quarterly*), which we considered highly relevant to our meta-analysis. We also applied a snowball procedure by browsing through several reference lists of existing research, specifically the reference lists of reviews (e.g., Grabe et al., 2008; Ward, 2016). We considered published and unpublished papers (i.e., conference papers, dissertations), and this search led to an initial sample of 622 papers.

### Selection of Papers

We applied three consecutive steps to narrow our list to those papers that were relevant for the meta-analysis. First, the first author excluded all qualitative research, theoretical research, content analyses, methodological research, narrative reviews, book reviews, commentaries, and research unrelated to the topic (e.g., anthropology, semiotics, art) by reviewing the title and the abstract of each paper. In this first step, we excluded 309 papers.

In the second step, we applied three inclusion criteria, which are relevant to the measure of media use, the measure of self-objectification, and the media content. All three variables are fully explained below as part of the analyses of moderators: (1) In previous studies, participants were asked about, not only their self-reported media use, but also their perception of being pressured by media to conform to existing beauty standards (e.g., Sociocultural Attitudes Towards Appearance Scale-3; Thompson, van den Berg, Roehrig, Guarda, & Heinberg, 2004). However, we were only interested in the direct link of media use and self-objectification; thus, we included only those studies that collected data on participants’ amount of time and frequency using a medium. We included only experimental studies that presented a media stimulus in both the experimental condition and the control condition. (2) Self-objectification had to be the dependent variable in experimental studies. In correlational studies, self-objectification had to be assessed as one of the investigated variables. (3) The experimental studies had to contain groups exposed to either sexualizing content or appearance-focused media content. When the experimental group was exposed to general media content only, the respective effect size was not coded and not included in the analysis. The control condition could include either nonsexualizing images (i.e., no or very few sexualizing references) or no people at all. With this second step, we excluded 240 papers.

In the third and last step, we excluded all papers that described an intervention (e.g., Choma et al., 2010; Harrison & Hefner, 2014; Veldhuis, Konijn, & Seidell, 2014). This subsumed any study that aimed at counteracting effects of media-induced self-objectification (e.g., presenting media literacy material before media exposure). Some intervention studies collect baseline (i.e., pre-intervention) data on media use and trait measures (e.g., self-objectification) in order to characterize their sample more fully or to consider moderators in the analysis of intervention effects. These data would have been relevant for our analysis. However, most of the interventional studies in our sample did not apply a pre-post design but used a post-only method instead. Other interventional studies did not measure media use at Time 1 (*t*1) and some studies did not report on the possible correlations. Thus, no correlations relevant to the meta-analysis were available and we excluded all interventional study designs from our sample.

We did not include papers that were not accessible (not available online) or that did not provide statistical information necessary for calculating the effect sizes. We contacted eight authors to get a copy of their dissertations and two authors to obtain additional statistical information; five authors did not respond and we had to omit five papers due to missing data. We also omitted all duplicates. That is, some papers were available as a dissertation and as published paper(s) or as conference papers and as published papers. In all but one of these cases, we coded the published papers. The exception was the paper by Aubrey and Taylor; we decided to code the conference paper (Aubrey & Taylor, 2005) instead of the published paper (Aubrey & Taylor, 2009) because it provided more effect sizes for the meta-analysis. The third and last step led to the exclusion of 19 papers.

### Final Sample of Studies

Our final sample included 54 papers. These papers yielded 50 independent studies (i.e., independent samples) with a total of 15,100 participants. Our sample consisted of articles from 27 journals, 4 conference papers, and 2 dissertations. Table 1 provides an overview of the included studies and the variables in the meta-analysis. The number of studies was smaller than the number of papers since there were several papers that relied on the same sample.^2^ We considered the results of such papers as derived from the same study; that is, we coded their effect sizes and subsequently treated them as stemming from a single study (Guo, 2016). Our sample size and the total number of participants were suitable for running a meta-analysis (see Pigott, 2012).

**Table 1.** Overview of the Coded Papers Including Aggregated Effect Size Estimates and Moderator Variables.

Study	*k*	*n*	*Zr*	Age	Female %	Ethnicity	Student Sample	Measure	Study Design	Media Content	Media Type	Study Location
Andrew, Tiggemann, and Clark (2016)	2	266	0.12	20.04	100.0	88.8	Yes	OBCS	CC	Online, print, TV	Sexualizing, general	AO
Arroyo and Brunner (2016)	5	488	0.10	20.51	66.2	73.6	Yes	OBCS	CC	Online	General	NA
Aubrey (2006a, b); Aubrey and Taylor (2005)	54	226	0.05	19.93	65.9	70.2	Yes	SOQ, OBCS	CP	Print, TV	Sexualizing	NA
Aubrey (2007)	4	384	0.16	19.60	59.1	70.8	Yes	SOQ, OBCS	CC	Print, TV	Sexualizing	NA
Aubrey (2010)	1	103	0.01	20.11	100.0	77.7	Yes	TST	E	Print	Appearance	NA
Aubrey and Gerding (2015)	1	94	0.23	20.05	100.0	56.8	Yes	TST	E	TV	Sexualizing	NA
Aubrey et al. (2009)	3	154	0.07	19.72	100.0	84.4		TST	E	Print	Sexualizing	NA
Barzoki, Mohtasham, Shahidi, and Tavakol (2016)	1	381	0.17	31.60	100.0			OBCS	CC	Online, TV	General	A
Dakanalis et al. (2012)	2	255	0.57	20.84	0.0		Yes	OBCS	CC	Print, TV	Sexualizing	E
Daniels (2009)	4	575	0.25	16.54	100.0	49.7		TST	E	Print	Sexualizing	NA
de Vries and Peter (2013)	2	221	0.17	20.80	100.0		Yes	TST	E	Print	Sexualizing	E
Doornwaard et al. (2014)	4	1,132	0.45	13.95	47.3			OBCS	CP	Online	Sexualizing, general	E
Fardouly, Diedrichs, Vartanian, and Halliwell (2015)	5	150	0.18	20.52	100.0		Yes	SOQ	CC	Online, print, TV	Sexualizing, general	E
Ford, Woodzicka, Petit, Richardson, and Lappi (2015, study 1)	2	99	0.16	19.90	49.5	85.9	Yes	SOQ	E	TV	Sexualizing	NA
Ford et al. (2015, study 2)	2	57	0.28	35.73	100.0	75.4	Yes	TST, SOQ	E	TV	Sexualizing	NA
Fox and Rooney (2015)	1	800	0.13	29.90	0.0	73.1	Yes	SOQ	CC	Online	General	NA
Fox et al. (2013)	1	86	0.30	21.16	100.0	53.5	Yes	TST	E	Video games	Sexualizing	NA
Fox et al. (2015, study 1)	1	87	0.32	20.04	100.0	79.3	Yes	TST	E	Video games	Sexualizing	NA
Fox et al. (2015, study 2)	1	81	0.29	19.91	100.0	67.9	Yes	TST	E	Video games	Sexualizing	NA
Grabe and Hyde (2009)	1	195	0.22	13.20	100.0	89.4		OBCS-Y	CC	TV	Sexualizing	NA
Grey, Horgan, Long, Herzog, and Lindemulder (2016)	2	66	0.61	18.97	100.0	47.0	Yes	TST	E	Print	Sexualizing	NA
Halliwell, Malson, and Tischner (2011)	3	122	0.13	19.98	100.0		Yes	SOQ	E	Print	Sexualizing	E
Harper and Tiggemann (2008)	2	90	0.24	20.48	100.0			TST	E	Print, TV	Sexualizing	AO
Harrison and Fredrickson (2003)	4	152	0.12	13.10	100.0	30.0		TST	E	TV	Appearance	NA
Hofschire (2003)	2	299	0.18	20.00	100.0	100.0	Yes	SOQ	CC	Print, TV	Sexualizing, general	NA
Hopper and Aubrey (2013)	3	301	0.11	29.20	100.0	92.2		TST	E	Print	Sexualizing, appearance	NA
Hopper and Aubrey (2016)	4	127	0.14	20.00	100.0	78.0	Yes	TST, OBCS	E	Print	Sexualizing, appearance	NA
Kalodner (1997)	2	103	−0.01	18.97	58.3	86.4	Yes	BSC	E	Print	Sexualizing	NA
Karsay and Matthes (2015)	1	151	0.15	23.98	100.0		Yes	TST	E	TV	Sexualizing	E
Kim et al. (2015)	2	562	0.18	20.63	100.0		Yes	OBCS, face-surveillance	CC	Print, TV	Sexualizing	A
Manago, Ward, Lemm, Reed, and Seabrook (2015)	6	815	0.22	19.07	57.3	73.8	Yes	OBCS-Y	CC	Online	General	NA
Meier and Gray (2014)	3	103	0.17	15.40	100.0	84.5		SOQ	CC	Online	Appearance, general	NA
Melioli, Rodgers, Rodrigues, and Chabrol (2015)	1	289	0.02	21.94	100.0		Yes	OBCS	CC	Online	General	E
Michaels, Parent, and Moradi (2013)	2	140	0.03	19.41	0.0	58.0	Yes	OBCS	E	Print	Sexualizing	NA
Miller (2007)	1	97	−0.02	15.97	100.0	81.4		OBCS	E	Print	Sexualizing	NA
Morry and Staska (2001)	4	150	0.17	19.30	59.3		Yes	SOQ	CC	Print	Sexualizing, general	NA
Nabi (2009, study 1)	2	170	0.03	22.00	56.0		Yes	OBCS	CC	TV	Sexualizing, general	NA
Nabi (2009, study 2)	2	271	−0.06	20.00	100.0	69.0	Yes	OBCS	CC	TV	Sexualizing, general	NA
Nowatzki and Morry (2009)	1	207	0.29	18.95	100.0	86.0	Yes	SOQ	CC	Print, TV	Sexualizing	NA
Prichard and Tiggemann (2012)	2	184	0.30	18.90	100.0	100.0	Yes	SOQ	E	TV	Sexualizing	AO
Tiggemann and Slater (2013), Slater and Tiggemann (2015)	10	1,087	0.21	13.70	100.0			SOQ, OBCS-Y (S)	CC	Online, print, TV	Sexualizing, general	AO
Tiggemann and Slater (2014)	6	189	0.25	11.50	100.0			OBCS-Y	CC	Online, print, TV	Sexualizing, general	AO
Tiggemann and Slater (2015)	11	204	0.17	11.64	100.0			OBCS-Y	CC	Online, print	Sexualizing, general	AO
Tylka (2015)	1	359	0.26	20.49	0.0	82.2	Yes	OBCS	CC	Online, print	Sexualizing	NA
Vandenbosch and Eggermont (2012, 2013, 2014, 2015a, 2015b)	76	1,041	0.16	14.73	43.4			SOQ, OBCS-Y	CC, CP	Online, print, TV	Sexualizing, appearance, general	E
Vandenbosch, Driesmans, et al. (2015)	4	75	0.32	13.35	49.3			SOQ	E	Video games	Sexualizing	E
Vandenbosch, Muise, et al. (2015)	1	495	0.23	20.26	100.0		Yes	SOQ	CC	TV	Sexualizing	E
Volgman (2014)	2	151	0.11	19.34	100.0	66.2		SOQ, OBCS	CC	Music	Sexualizing	NA
Ward, Seabrook, Manago, and Reed (2015)	3	1,107	0.12	19.19	59.5	72.9	Yes	OBCS-Y	CC	TV	Sexualizing	NA
Zurbriggen, Ramsey, and Jaworski (2011)	1	159	0.06	19.08	57.2	67.9	Yes	OBCS	CC	Print, TV	Sexualizing	NA

*Note*. *k* = number of obtained effect sizes*; n* = number of participants on which the effect size was based on; *Zr* = effect size, Fisher *r*-to-*z* transformed; A = Asia*;* AO = Australia and Oceania; E = Europe; NA = North America; SOQ = Self-Objectification Questionnaire; TST = Twenty Statements Test; OBCS = Surveillance subscale of the Objectified Body Consciousness Scale; OBCS-Y = Surveillance subscale of the Objectified Body Consciousness Scale–Youth; BSC = Public Body-Consciousness subscale of the Body Self-Consciousness Questionnaire; CC = Correlational Cross-Sectional Study; CP = Correlational Panel Study; E = Experiment.

### Moderator Variables

We were interested in whether sample or study design characteristics would moderate the postulated relation between sexualizing media use and self-objectification. Our analysis of possible moderators was limited to those that (a) were theoretically relevant, (b) provided a sufficient number of effect sizes, and (c) showed sufficient variance to test the moderation. For instance, we included gender as a moderator because objectification theory (Fredrickson & Roberts, 1997) explains why women face more objectifying experiences in their daily lives than men. Thus, larger effect sizes for self-objectification might be expected for women compared to men. Higgins and Green (2011) suggested considering moderator analysis only if there were 10 or more studies that incorporated the moderators. For categorical moderators (e.g., media type), only those moderator categories present in at least two different studies were included. We differentiated between moderators with regard to sample characteristics and study design characteristics.

### Sample Characteristics

We investigated whether or not the age of the participants moderated the results by coding the mean age. And we included the gender distribution within each sample, which was coded as male (0), mixed (1), or female (2), as a moderator. Ethnicity, the percentage of White or Caucasian participants, was coded for all studies conducted in the United States. We also included a dichotomous variable that indicated whether participants were predominantly students (1) or not (0).

### Study Design Characteristics

We included the following six moderator variables for study design characteristics:

#### Measure of self-objectification

Based on methodological reflections (Calogero, 2011; Moradi & Huang, 2008) and the meta-analysis by Grabe et al. (2008), we included the most common measures of self-objectification. We coded the TST (1) and modified versions of the TST that followed the same principle of listing appearance-related (as opposed to nonappearance-related) self-descriptions. We also coded the SOQ (2), the Surveillance subscale OBCS (3), the Surveillance subscale of the Objectified Body Consciousness Scale–Youth (4; OBCS-Y; Lindberg, Hyde, & McKinley, 2006), the Public Body-Consciousness subscale of the Body Self-Consciousness Questionnaire (5; BSC; L. C. Miller, Murphy, & Buss, 1981), and other (= face surveillance; 6). We included BSC because the scale assesses sense of self-consciousness in application to the body and thus strongly reflects self-objectification (McKinley & Hyde, 1996). We coded one study that used the face surveillance scale (Kim et al., 2015) because it represented a culture-specific form of self-objectification.

#### Design type

We coded the study design type as experimental design (0), cross-sectional survey (1), or panel survey (2). We coded effect sizes from experimental studies as experimental design; effect sizes that reflected survey data from 1 point in time (e.g., sexualizing media use *t*1 and self-objectification *t*1) were coded as cross-sectional survey; effect sizes that reflected survey data from 2 points in time, that is, cross-lagged data (e.g., sexualizing media use *t*1 and self-objectification Time 2 [*t*2]), were coded as panel survey.

#### Media type

We wanted to know whether or not the type of medium moderated the effect of media use on self-objectification. We coded overall television use, the use of specific television programs or shows (e.g., sitcoms, music videos), and the presentation of audiovisual material in experimental studies (e.g., video clips, television advertisements) in the television (0) category. When the use of print media was examined or when participants were exposed to photographs or print advertisements in experiments (even if the study was conducted online), we coded the medium as print (1). Using the Internet or social networking sites was coded as online (2). We coded watching or playing a video game as video game (3). Listening to music was coded as music (4).

#### Media content

We assessed the media content as sexualizing and appearance focused (0), appearance focused (not sexualizing; 1), or general (2). To avoid confusion, we refer in the remainder of the article to the first category as “sexualizing.” We identified media content as sexualizing when it matched the APA (2007) definition of sexualization. To code experimental studies, we carefully read the description of the stimulus and, if provided, looked at pictures of the stimulus material. For correlational studies, we defined the following media as sexualizing: pornography, the so-called “lad media” (i.e., media specifically targeted at a male audience such as *Maxim* or *FHM*), music videos, music television, reality television, and fashion, beauty, and youth magazines (APA, 2007; Klaassen & Peter, 2015; Stankiewicz & Rosselli, 2008; Vandenbosch et al., 2013). In some correlational studies (e.g., Aubrey, 2006a, 2006b; Vandenbosch & Eggermont, 2013), the authors applied a procedure in order to attribute more weight to media considered more sexualizing. Respondents first indicated their use of several media types and genres. After data collection, an independent jury rated the media in regard to frequency and intensity of sexualization. Based on the jury assessment, a sexualization score was calculated for each medium and applied to weight the media measures (for a further description of the procedure, see Zurbriggen, Ramsey, & Jaworski, 2011). We treated the weighed media measures as sexualizing media content. Some researchers included in their studies media content that was neither sexualizing nor general (e.g., Aubrey, 2010; Harrison & Fredrickson, 2003; Meier & Gray, 2014) but was still relevant to the study. We accounted for this nonsexualizing media content by defining it as appearance-focused (Moradi, 2010; Vandenbosch & Eggermont, 2015a). For instance, watching or posting photos on Facebook (Meier & Gray, 2014) was categorized as appearance-focused content. Experimental conditions that expose participants to articles with an appearance frame, as distinct from a health frame, were coded as appearance-focused content (Aubrey, 2010). Finally, we defined the general use of the Internet, social networking sites, or television, as well as using news and sports media, as exposure to general media content.

#### Study location and year of publication

We coded the study location based on the continent in which the study was conducted: North America (1), Europe (2), Asia (3), and Australia and Oceania (4). If the continent or country was not explicitly mentioned, the authors’ affiliation served as an indicator. And we included the year of print publication as a potential moderator in the analysis.

#### Intercoder reliability

In order to assess inter-coder reliability, two coders (first and second author) coded a subsample of 36 effect sizes. Krippendorff’s (2004) α was perfect (α = 1.0) for all variables, except for the moderator measurement of self-objectification (α = .92). Discrepancies were resolved through discussion after reviewing the concerned study. Afterward, the two coders coded all variables based on the information available in the manuscripts.

### Statistical Model and Effect Size Calculation

#### Statistical model

Several studies reported results that enabled us to code more than one effect size per study. Performing a meta-analysis on these studies would violate the assumption of independence of effect sizes and assign more weight to the studies producing more than one effect size. Researchers recently suggested treating meta-analysis as a multilevel model to address these issues (e.g., Cheung, 2014; Field, 2015; Konstantopoulos, 2011). The basic idea nests the effect size (first level) within the studies (second level; Konstantopoulos, 2011; for more detailed information, see Field, 2015). Effect sizes stemming from the same study receive the same random effect, whereas effect sizes stemming from different studies receive different random effects. Hence, the dependence or independence of effect sizes is explicitly modeled by assigning the correct random effect (Konstantopoulos, 2011; Viechtbauer, 2015). Consequently, all effect sizes can be taken into account without aggregation and loss of information. This procedure is especially valuable when it comes to moderator analysis because multiple effect sizes within studies are usually connected to different levels of a moderator variable. Results were comparable when calculating simple instead of multiple regression models.

We coded the following information for each paper: (a) all effect sizes, including group differences, means, standard deviations, and standard errors in experimental research. If several conditions matched the requirements for a control group, we included effect sizes for each control group. In correlational studies, we coded Pearson’s *r*; if correlational studies were panel surveys, we coded all available effect sizes, as long as self-objectification was not preceding media use (i.e., media use *t*1 and self-objectification *t*1, media use *t*1 and self-objectification *t*2, and media use *t*2 and self-objectification *t*2 were coded). And we coded (b) all moderators.

#### Effect size calculation

We used Pearson’s *r* as the effect size estimate because it can be easily interpreted in terms of its practical importance. Its size ranges finitely from 0 to 1 (Rosenthal & DiMatteo, 2001). A positive *r* indicates that as media use increases, self-objectification increases. In correlational studies, we took *r* directly from the articles. In one case (Doornwaard et al., 2014), we coded the standardized regression coefficient instead, and we transformed it to *r* according to the formula provided by Peterson and Brown (2005). In experimental studies, we calculated *r* according to the formulas provided by Lipsey and Wilson (2001). Before performing the syntheses, we converted the correlation coefficients (*r*) to the Fisher’s *z* scale (*Zr*; Borenstein, Hedges, Higgins, & Rothstein, 2009; Lipsey & Wilson, 2001). In total, we obtained 261 effect sizes.

We carried out the meta-analysis by using the R metafor package (Viechtbauer, 2010). We based the estimates on random-effects models. Random-effects models assume differing true effect sizes vary, for instance, because of different participants or treatments. In addition, random-effects results may be generalized beyond the studies included in the analysis because the investigated studies are treated as a random subset of a larger study population (Hedges & Vevea, 1998). The moderator analyses were carried out using the rma.mv() function of the R metafor package, which enabled the estimation of multilevel mixed-effects models (Viechtbauer, 2010). We performed the overall effect and publication bias analyses with effect sizes aggregated within studies using the rma() function. This approach enabled the estimation of single-level random-effects models (Viechtbauer, 2010; see Pearce & Field, 2016, for a similar approach). We applied a maximum likelihood estimator.

As studies showed considerable variance in sample size, and some produced multiple effect size estimates, we weighted the effect sizes by sample size and the number of effect sizes per study. Larger and therefore more precise studies received greater weight. And studies reporting multiple effect sizes did not receive more weight than studies reporting only one effect size. Accordingly, we weighted effects sizes by computing the ratio of the study’s sample size to the number of effect sizes coded from the study (Hunter & Schmidt, 2004). For instance, if Study 1 had 200 participants and yielded one effect size, this effect size was assigned a weight of 200/1 = 200. If Study 2 had 200 participants and yielded four effect sizes, each of the effect sizes was assigned a weight of 200/4 = 50. Calculating the mean effect size, Study 1 received a weight of 200, while Study 2 received a weight of 4 × 50, resulting in the same overall weight.

## Results

### Overall Effect Analysis

Table 1 presents all individual effect sizes. The overall effect analysis revealed a positive, small to moderate effect of media use on self-objectification (*r* = .19, *Zr* = .19). The effect was significant, 95% CI [.15, .23], *p* < .0001. Following Rosenthal (1979), we calculated the so-called file drawer analysis, which addressed the concern that there may be additional studies not included in the analysis that failed to be published because their effect size was zero, or at least considerably smaller. Including them in the analysis may have possibly resulted in a nonsignificant overall effect (Borenstein et al., 2009). To address this concern, Rosenthal (1979) suggested an approach to calculate the number of zero-effect studies needed to nullify the found result (Borenstein et al., 2009). The analysis revealed a fail-safe *N* of 7,816. Thus, the observed effect is highly robust.

In addition, we found significant heterogeneity among effect sizes, *Q*(49) = 213.72, *p* < .0001. This suggests that effect sizes vary considerably due to between-study differences. The *I* ^2^ statistic—the amount of total variability (sampling variance + heterogeneity) that can be attributed to the heterogeneity among the true effects (Higgins & Thompson, 2002)—provided further insights. About 75% of the total variability can be attributed to between-study differences (*I* ^2^ = 75.03). It seemed likely that our moderators might explain some of these differences (Huedo-Medina, Sánchez-Meca, Marín-Martínez, & Botella, 2006).

### Moderator Analysis

We tested the moderated effects by calculating meta-regressions (multilevel mixed-effects model). For each moderator, we calculated a separate meta-regression. Categorical moderators (i.e., gender, measure, design type, media type, media content, and study location) were dummy coded. We treated the most frequently coded categories as the reference categories. Regression coefficients represent changes in effect size according to changes in moderator levels. The χ^2^ test statistic indicated whether a moderator, taken as a whole, significantly affected effect size (*Q* test; Borenstein et al., 2009). In contrast, the *z* test statistic indicated whether or not a certain level of categorical moderator was significantly different from the reference category of this moderator (*Z* test; Borenstein et al., 2009). Tables 2 and 3 display all results.

Looking at Table 2 (sample characteristics), there were no significant moderation effects. That is, the effect of media use on self-objectification appeared to be independent of participants’ age, gender, and ethnicity, as well as independent of whether or not participants were students.

**Table 2.** Meta-Regression Results for Testing the Influence of Sample Characteristics on Effect Size.

					95% CI		
Change in Effect Size If		*N*	*k*	Estimate	LL	UL	Test
Mean age:	Increases by 1 year	50	261	−.01	−.02	.002	χ^2^(1) = 2.68
Percentage of female participants (gender):	Increases by 1%	50	261	−.0001	−.001	.001	χ^2^(1) = .07
Percentage of Caucasian participants (ethnicity):	Increases by 1%	30	121	.0003	−.003	.003	χ^2^(1) = .04
Use of student samples:	Student compared to nonstudent	50	261	−.06	−.14	.02	χ^2^(1) = 1.95

*Note. N* = number of independent studies included in the respective regression; *k* = number of effect sizes included in the respective regression; estimate = meta-regression coefficients for *Zr*; CI = confidence interval with lower (LL) and upper limit (UL); χ^2^ = test statistic of *Q* test.

^†^ *p* < .10. *p < .05.

Looking at Table 3 (study design characteristics), media type moderated effect size significantly, χ^2^(3) = 7.65, *p* = .05. The effect size *Zr* was .11 (*z* = 2.13, *p* < .05), indicating a stronger effect when participants used online media instead of television. In addition, the effect size was .18, stronger when participants used video games instead of television (*z* = 2.24, *p* < .05). The use of print media did not lead to any differential effects, neither when compared to television nor when compared to online media or video games. The remaining study design characteristics did not impact effect size. That is, the effect of media use on self-objectification appeared to be independent of the type of measurement of self-objectification, the study design, and the media content. There was a trend indicating that the study location moderated effect size, χ^2^(3) = 6.60, *p* = .09. Specifically, the effect size *Zr* of European studies was .12 larger when compared to studies from North America (*z* = 2.53, *p* < .05). In contrast, neither Asian nor Australian studies differed significantly from North American studies, nor did they differ from European studies. Year of publication did not moderate the overall effect size.

**Table 3.** Meta-Regression Results for Testing the Influence of Study Design Characteristics and Year of Publication on Effect Size.

				95% CI		
Change in Effect Size If	*N*	*k*	Estimate	LL	UL	Test
Measure:	49	258				χ^2^(3) = .43
TST instead of SOQ			.02	−.08	.13	*z* = .43
OBCS instead of SOQ			.03	−.07	.13	*z* = .61
OBCS-Y instead of SOQ			.002	−.10	.10	*z* = .05
Design type:	50	261				χ^2^(2) = .35
Experiment instead of cross-secondary survey			−.01	−.09	.07	*z* = −.36
Panel instead of cross-secondary survey			−.04	−.21	.13	*z* = −.49
Media type:	41	245				χ^2^(3) = 7.65*
Print instead of television			.05	−.03	.13	*z* = 1.14
Online instead of television			.11	.01	.20	*z* = 2.13*
Video game instead of television			.18	.02	.33	*z* = 2.24*
Media content:	50	261				χ^2^(2) = .59
Appearance-focused instead of sexualizing			−.03	−.13	.07	*z* = −.68
General instead of sexualizing			−.02	−.09	.06	*z* = −.46
Study location:	50	261				χ^2^(3) = 6.60^†^
Europe instead of North America			.12	.03	.22	*z* = 2.53*
Asia instead of North America			.02	−.13	.17	*z* = .30
Australia instead of North America			.06	−.06	.17	*z* = .91
Year of publication:						
Increases by 1 year	50	261	.06	−.06	.17	*z* = 0.91

*Note. N* = number of independent studies included in the respective regression; *k* = number of effect sizes included in the respective regression; estimate = meta-regression coefficients for *Zr*; CI = confidence interval with lower (LL) and upper limit (UL); χ^2^ = test statistic of *Q* test; *z* = test statistic of *Z* test; SOQ = Self-Objectification Questionnaire; TST = Twenty Statements Test; OBCS = Surveillance subscale of the Objectified Body Consciousness Scale; OBCS-Y = Surveillance subscale of the Objectified Body Consciousness Scale–Youth.

^†^ *p* < .10. *p ≤ .05.

We also checked for interaction effects between moderators. Specifically, we assumed that men and women (gender), younger and older participants (age), or students and nonstudents (student sample) would respond differently to sexualizing, appearance-focused, and general media content (content). However, there were no significant interactions between the type of content and one of the three moderators: Gender × Content: χ^2^(2) = .12, *p* = .94; Age × Content: χ^2^(2) = .30, *p* = .86; Student Sample × Content: χ^2^(2) = 1.02, *p* = .60. In conclusion, the effect of media use on self-objectification appeared to be very robust. Besides the effect of study location and media type, self-objectification was unaffected by the analyzed boundary conditions.

### Publication Bias Analysis

**Figure 2.**
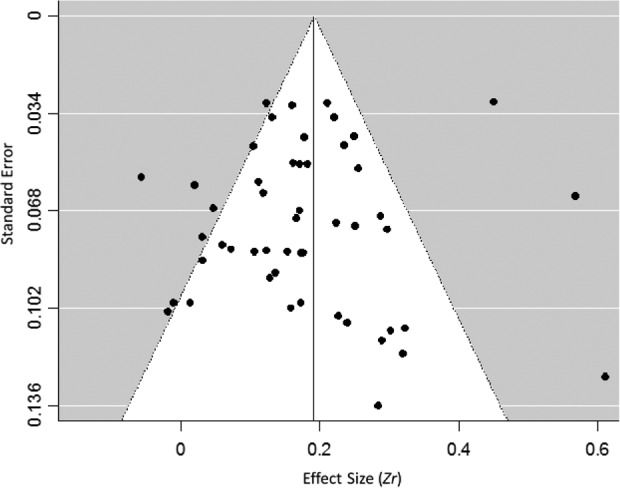
Funnel plot of the studies in the meta-analysis.

Last, we checked for publication bias. We tested whether or not studies with small samples and minor effect sizes failed to be published. We applied a funnel plot and Egger’s regression test for funnel plot asymmetry (Egger, Smith, Schneider, & Minder, 1997). As recommended in the literature, we used the standard error as an indicator of sample size (Borenstein et al., 2009). Looking at the funnel plot (Figure 2), there was slight evidence of publication bias in terms of smaller studies with minor effect sizes missing at the bottom left corner. However, this pattern was reversed when looking at the middle part of the figure (studies with major effect sizes missing), arguing against publication bias. Furthermore, a nonsignificant Egger’s regression test, *t*(48) = −1.00, *p* = .33, indicated that publication bias was not confirmed.

## Discussion

Self-objectification is an increasingly important concept in media effects research. Stimulated by the work of objectification theorists (e.g., Fredrickson & Roberts, 1997; McKinley & Hyde, 1996), in many empirical studies, scholars have investigated the influence of sexualizing media on self-objectification. Based on a meta-analysis that included 50 studies (261 effect sizes), encompassing three types of research designs, we were able to show in the current study that, across various types of mass media presenting varying degrees of sexualizing content, there is a positive effect of sexualizing media use on self-objectification (*r* = .19). As hypothesized, the use of mass media increased self-objectification among women and men. The effect was very robust and small to moderate in terms of size (Lipsey & Wilson, 2001).

### Sample Characteristics

None of the sample characteristics (age, gender, ethnicity, and student sample) moderated the main effect. Fredrickson and Roberts (1997) posited that women of all age groups are potentially objectified. It can be argued, however, that younger individuals are more susceptible to sexualizing media content (Fortenberry, 2013). Yet our meta-analysis showed no moderation effect of the mean age and did not support this assumption. It has to be noted, however, that the age range of our samples was quite truncated, consisting almost entirely of adolescents and emerging adults. We discuss this issue further in the Limitations section.

Furthermore, we found no moderation of gender on the effect of sexualizing media use on self-objectification. A possible explanation is that the media environment has changed. Findings from past content analysis research have indicated that men face the increasing probability of encountering sexualized depictions of men (Gill, 2009; Hatton & Trautner, 2011; Ricciardelli, Clow, & White, 2010; Rohlinger, 2002). Although sexualization of men and women have different social meanings, in the end, the sexualized body becomes an object that is disciplined, manipulated, and scrutinized by others (Rohlinger, 2002), leading to self-objectification among both women and men. This may explain why men, compared with women, showed similar effects of sexualizing media use on self-objectification. Our result corroborates earlier research that identified gender similarities in the relation between self-objectification and body esteem or body shame (Moradi & Huang, 2008). However, we must consider the implications of different cultural standards that are applied to women and men. The cultural ideal for male attractiveness includes strength, muscularity, and dominance, whereas the cultural ideal for female attractiveness revolves around thinness and vulnerability (Moradi, 2010). Thus, our finding should not obscure the fact that existing power relations and discriminations are perpetuated (Moradi, 2010). In addition, women tend, in the course of development across the life span, to receive more sexualizing information, comments, or actions than men (e.g., Swim, Hyers, Cohen, & Ferguson, 2001).

We found no moderation effect of participants’ ethnicity. The studies we included allowed us to differentiate only between White/Caucasian and any non-White/other ethnicities. Grouping different ethnicities together may result in overlooking differences that may exist because one group could be canceling out the effects of another. For instance, a longitudinal study has shown that African American girls reported less body dissatisfaction across high school years relative to other girls. Asian girls, however, reported increased body dissatisfaction when compared to African American girls, Latina girls, and multiethnic girls (de Guzman & Nishina, 2014). However, a meta-analysis on ethnicity and body dissatisfaction that included Asian, American, Black, Hispanic, and White women found only a small difference in greater body dissatisfaction for White women when compared to Black women (Grabe & Hyde, 2006). Another explanation may be found in the media content. Previous researchers have noted that Black women prefer silhouettes representative of a curvier body rather than the prevalent thin-ideal presented in the media (Capodilupo & Kim, 2015; Overstreet, Quinn, & Agocha, 2010). The lack of representation of minority women in the media might create similar results in women of color and White women, as neither group is exposed to images accurately representing them. This issue is further discussed in the section on future research.

### Study Characteristics

We found that the use of video games and/or online media led to stronger self-objectification effects when compared to television use. Several explanations can be considered for this effect. Both media types are characterized by relatively high levels of interactivity and control (Eveland, 2003). In other words, whereas one can easily watch television and do something unrelated at the same time, this is more difficult with video games and, to some degree, also more difficult with online media. Video games may lead to high levels of the psychological experience of presence, namely, the feeling of being located in a media environment (Weibel, Wissmath, & Mast, 2011; Wirth et al., 2007). Furthermore, video games are known for highly sexualized depictions of female and male game characters (e.g., Burgess et al., 2007; Lynch, Tompkins, van Driel, & Fritz, 2016), and many games enable individuals to play a character with a different body, possibly a more idealized body type than the player’s own body type. Social networking sites are online media that are characterized by their personalized, visual content revolving around the self. Idealized videos and pictures of the self, peers, and other individuals might foster social comparisons and the internalization of appearance ideals and, in turn, might increase self-objectification among individuals (Perloff, 2014).

We found no significant moderation effect for the type of measures of self-objectification. On the one hand, this result suggests that all measures included in the current study appeared to be equally effective in capturing media effects on self-objectification. On the other hand, it can be speculated that the effect of sexualizing media is equally strong for cognitive and behavioral aspects of self-objectification, since cognitive measures (e.g., SOQ) and behavioral measures (e.g., OBCS subscale) were included in the analysis. However, researchers have demonstrated that self-objectification and body surveillance are related to each other but are not equal (Calogero, 2011; Moradi & Huang, 2008). Further research is needed in order to draw final conclusions about the effect of sexualizing media on and differences between existing measures of self-objectification.

We identified no significant effect for design type: cross-sectional survey studies, panel survey studies, and experimental studies yielded similar results; that is, we identified no statistically significant differences in effect sizes. Media content also had no moderating effect. Most studies included here investigated exposure to sexualizing media content. Thus, we can assume that this specific kind of content may lead to self-objectifying thoughts or behavior. However, appearance-focused (nonsexualizing) and general media content also predicted self-objectification in our study. This nonsignificant moderation may be explained by cultivation theory (e.g., Gerbner, 1998). The pervasive presence of sexualizing content in all kinds of mass media (e.g., Aubrey & Frisby, 2011; Burgess et al., 2007; Lynch et al., 2016; Stankiewicz & Rosselli, 2008; Vandenbosch et al., 2013) may have a cumulative and mutually reinforcing effect on self-objectification among individuals. However, the assumption of homogeneous mass media effects has been criticized (e.g., Bilandzic & Rössler, 2004). Results from related media effects research indicated that the use of specific media content predicted body dissatisfaction, whereas total media consumption did not (Levine & Murnen, 2009; Meier & Gray, 2014). In line with this reasoning, Andrew, Tiggemann, and Clark (2016) have recently shown that the use of nonappearance media, like information-based shows, documentaries, and news, was negatively related to self-objectification. Thus, we do not believe that any media content will automatically lead to self-objectification (Levine & Murnen, 2009). Rather, media that focus—to some extent—on outward appearance should have an influence. Furthermore, we believe that the lack of moderation by media content may reflect limits in the methods used and types of data gathered in the analyzed studies. We discuss this issue more thoroughly in the section on Limitations.

We found a slight tendency for study location as a moderator: The effect for European studies was higher when compared to studies from North America. However, it is possible that this effect stemmed mostly from the study by Doornwaard et al. (2014). The Doornwaard et al. (2014) study was among the very few that investigated the effects of highly explicit sexualizing content, namely, pornography. Moreover, the large sample (*N* = 1132) of adolescents that Doornwaard et al. (2014) used in their study gave more weight to their effect sizes in our analysis. When running the moderator analysis without the study, the moderation effect of study location was not significant, which supports our explanation.

In sum, our findings suggest that the effect of sexualizing media use on self-objectification is very robust. It is important to stress that we found almost no effects of these potentially intervening variables, even though the number of studies and the sample sizes were clearly sufficient to run moderator analyses.

### Limitations and Agenda for Future Research

In the following sections, we address limitations of the present study and research gaps in the field of body image research and media effects research and we provide an agenda for future research. In the present study, we included only papers that were available in English. However, the file drawer analysis indicated a highly robust effect. In addition, we are aware of the fact that coding the study location by continent may not sufficiently capture all the differences in objectification that may stem from individuals’ cultural origin; countries within each continent are likely to vary in their types of sexualizing images portrayed in the media (e.g., Collins, 2011). Finally, although we conducted a thorough literature search for the meta-analysis, we cannot rule out that single studies were missed, especially those that were unpublished or unavailable on the Internet. Nevertheless, we believe that this limitation does not diminish our findings as we applied a random effects model for the meta-analysis. Thus, in our analysis, the investigated studies were treated as a random subset of a larger study population (Hedges & Vevea, 1998). We also found no evidence for a publication bias.

The research field we examined also has limitations. These include shortfalls in regard to the investigated samples, the lack of longitudinal studies, and insufficiently investigated variables.

### Shortfalls of investigated samples

Our findings demonstrated that research on media and objectification conducted outside of Western or Westernized countries is scarce. Although this blatant bias has been pointed out before (Moradi & Huang, 2008), it is striking. Ninety-six percent (*n* = 48) of the investigated studies we identified originated from North America, Europe, or Australia and Oceania. Only two studies were from Asia (Barzoki, Mohtasham, Shahidi, & Tavakol, 2016; Kim et al., 2015), and none were from Latin America or Africa.

Furthermore, most studies on self-objectification focused on women. In our meta-analysis, two thirds (*n* = 33) of the studies exclusively investigated women. Women face more interpersonal sexualizing experiences compared to men (Swim et al., 2001), and women are more likely to be sexualized in a wide range of media types (Aubrey & Frisby, 2011; Burgess et al., 2007; Stankiewicz & Rosselli, 2008; Vandenbosch et al., 2013). And women commonly report higher levels of self-objectification than men (e.g., Aubrey, 2006a; Lindberg et al., 2006; Vandenbosch & Eggermont, 2015b; Ward, Seabrook, Manago, & Reed, 2015). However, our results suggest that the media effect on self-objectification is similar for both genders. Thus, it is important to include both women and men in self-objectification research.

Considering the fact that the average mean age of the investigated participants was 19.67 years, research among younger and older individuals is needed. Since sexualizing experiences and self-objectification begin at a very young age, researchers have recently investigated sexualization of, and self-objectification among children (e.g., E. Holland & Haslam, 2016; Jongenelis, Byrne, & Pettigrew, 2014; Slater & Tiggemann, 2016). It is equally important to include older populations because self-objectification might change over time (Fredrickson & Roberts, 1997).

Finally, research on different ethnicities is missing. For example, to the best of our knowledge, only one experimental study investigated the effects of media exposure on self-objectification among White girls and girls of color (Harrison & Fredrickson, 2003). It follows that future research should include both women and men in different stages of life outside the “Western bubble” in order to test the cross-cultural applicability of theoretical frameworks, like objectification theory (Moradi & Huang, 2008).

We suggest that in the future, researchers should investigate the extent to which children, adolescents, and/or emerging adults of differing ethnicities are exposed to differing amounts of sexualizing content. Furthermore, we recommend that researchers in a variety of countries, such as England, Germany, and Australia, need to be more careful and conscientious about gathering information concerning ethnicity.

### Lack of longitudinal studies

We identified an evenly distributed number of experimental designs and cross-sectional survey designs in the studies we included. However, there were few longitudinal survey studies; we identified only three independent samples that used this approach (Aubrey, 2006a, 2006b; Aubrey & Taylor, 2005; Doornwaard et al., 2014; Vandenbosch & Eggermont, 2014, 2015a, 2015b). More longitudinal research is needed in order to further determine prospective, and thus possibly causal, effects by estimating cross-lagged relations and intraindividual change in externally valid settings (G. Holland & Tiggemann, 2016; Valkenburg & Peter, 2013).

### Insufficiently investigated variables

Internalization of appearance ideals is a key variable that was not included in our analysis. We believe that it would be valuable to look into this concept more thoroughly. Fredrickson and Roberts (1997) explicitly refer to the internalization of appearance ideals as an explanatory mechanism leading to self-objectification. They and others have theorized that experienced or anticipated sexual objectification leads to an internalization of appearance ideals, which in turn results in self-objectifying thoughts or behavior (Fredrickson & Roberts, 1997; Moradi, 2010; Moradi & Huang, 2008). Researchers have repeatedly shown that internalization functions as a mediator between sexualizing media use and self-objectification (Tiggemann & Slater, 2014; Vandenbosch & Eggermont, 2012, 2013, 2014). However, other researchers have not found support for a mediating effect of internalization on self-objectification (Aubrey, 2006b; Karsay & Matthes, 2015). Research is needed on the internalization of appearance ideals in order to shed light on these contradictory findings.

In addition, the following two understudied variables should be explored in the future: socioeconomic status and gender role perceptions. However, these two variables do not represent an exhaustive list of understudied variables. Past research on body dissatisfaction has shown that high socioeconomic status is linked to body dissatisfaction and drive for thinness among women (Swami et al., 2010). Thus, it seems possible that socioeconomic status plays a role in self-objectification. In addition, within-gender differences, such as gender role perceptions, should be further investigated because hypergender orientation has been related to sexualizing media use, self-objectification, and sexualizing behaviors (Nowatzki & Morry, 2009; van Oosten, Peter, & Boot, 2015).

We also identified several insufficiently studied variables in regard to media use. Specifically, self-reported media use was inconsistently measured in correlational research. Whereas some studies assessed media use with differing nominal scales (e.g., Andrew et al., 2016; Fardouly et al., 2015), other studies included metric measures by asking participants about the specific amount of time they used a certain media type (e.g., Barzoki et al., 2016).

Empirical findings based on the media priming framework have shown that the intensity of a media prime influences the strength of the media effect (e.g., Arendt, 2013). Therefore, for the experimental studies, we initially coded the frequency and duration of participants’ exposure to media. However, many studies failed to report these data and the variance of the coded data was very low. Thus, we could not include the frequency and the duration of media exposure as a moderator in the final analysis. In addition, only very few studies have investigated the relation of sexually explicit media content and self-objectification (e.g., Tylka, 2015; Doornwaard et al., 2014), although it has been shown that pornographic content contains many objectifying depictions (Klaassen & Peter, 2015). These different (and absent) measures of media use might account for (a) the null and mixed results in the field and (b) the large variability of between-study differences we found in our meta-analysis. We suggest researchers look more closely at media content, genres, and titles when investigating the relation of media use and self-objectification. Furthermore, researchers should report the particular kind of content, genres, or titles they are studying (see also Valkenburg & Peter, 2013). This would help to understand which content influences self-objectification and which content does not. Future researchers might also investigate possible interaction effects between media type and media content. For example, video games are known for their sexualizing content (e.g., Burgess et al., 2007) and, at the same time, video games can lead to high levels of presence, which might lead to higher levels of self-objectification.

Finally, as Moradi and Huang (2008) have already stressed, it is important to distinguish between trait and state terminology when discussing self-objectification. Only 16 of the 50 studies indicated a distinction between trait and state self-objectification. Closely related to the measurement issue, other concepts linked to self-objectification should be considered in future research, such as Piran’s (2015, 2016) construct of disembodiment or Tolman and Porche’s (2000) objectified relationship with one’s body.

### Practice Implications

The results from the current meta-analysis can inform prevention and intervention efforts in clinical and educational contexts. For instance, therapists and counselors might encourage their clients to reflect on their use of sexualizing and appearance-focused media. Teaching institutions might pick up on the moderating effect of video games and online media to increase awareness among their students, since both media types are extremely popular among children and adolescents. Teachers and educators could teach students how to identify sexualizing and appearance-focused media content and explain the potential negative effects on self-objectification and other health-related issues, such as body shame, body dissatisfaction, and eating disorders. Both scholars and practitioners might work on intervention strategies to circumvent or mitigate media effects on self-objectification. Overall, practitioners and scholars who are involved in body image topics and women’s health can benefit from the review of the empirical literature and from the identification of an agenda for future research.

### Conclusions

We tried to quantify the effect of sexualizing media use on self-objectification by using a meta-analytic approach. The results showed a small to moderate overall effect. We found a moderation effect of media type, suggesting that the effect was more pronounced for participants using video games or online media. Moreover, the findings suggest that the effect of media use on self-objectification equally affected men and women, older and younger participants, and participants of several ethnic backgrounds. We call for future research to include both men and women in all life stages and from different parts of the world, to implement longitudinal designs, to further investigate the internalization of appearance ideals, and to report more extensively on the measures regarding media use. We hope that the findings of our study will stimulate researchers to address the outlined research gaps in their future research. Furthermore, we hope the article will encourage practitioners and parents to reflect on the role of sexualizing media use in the development of individuals’ self-objectification.

## Notes

1. Data may be obtained from the first author upon request.

2.
Aubrey (2006a), Aubrey (2006b), and Aubrey and Taylor (2005) are based on the same sample. Likewise, Tiggemann and Slater (2013) and Slater and Tiggemann (2015) are based on the same sample. Finally, Vandenbosch and Eggermont (2012), Vandenbosch and Eggermont (2013), Vandenbosch and Eggermont (2014), Vandenbosch and Eggermont (2015a), and Vandenbosch and Eggermont (2015b) are also based on the same sample.

## References

[bibr1-0361684317743019] References marked with an asterisk indicate studies included in the meta-analysis.

[bibr2-0361684317743019] American Psychological Association, Task Force on the Sexualization of Girls. (2007). Report of the APA task force on the sexualization of girls. Washington, DC: American Psychological Association Retrieved from http://www.apa.org/pi/women/programs/girls/report-full.pdf

[bibr3-0361684317743019] *Andrew R. Tiggemann M. Clark L. (2016). Predicting body appreciation in young women: An integrated model of positive body image. Body Image, 18, 34–42. doi:10.1016/j.bodyim.2016.04.003 27240100 10.1016/j.bodyim.2016.04.003

[bibr4-0361684317743019] Arendt F. (2013). Dose-dependent media priming effects of stereotypic newspaper articles on implicit and explicit stereotypes. Journal of Communication, 63, 830–851. doi:10.1111/jcom.12056

[bibr5-0361684317743019] *Arroyo A. Brunner S. R. (2016). Negative body talk as an outcome of friends’ fitness posts on social networking sites: Body surveillance and social comparison as potential moderators. Journal of Applied Communication Research, 44, 216–235. doi:10.1080/00909882.2016.1192293

[bibr6-0361684317743019] *Aubrey J. S. (2006a). Effects of sexually objectifying media on self-objectification and body surveillance in undergraduates: Results of a 2-year panel study. Journal of Communication, 56, 366–386. doi:10.1111/j.1460-2466.2006.00024.x

[bibr7-0361684317743019] *Aubrey J. S. (2006b). Exposure to sexually objectifying media and body self-perceptions among college women: An examination of the selective exposure hypothesis and the role of moderating variables. Sex Roles, 55, 159–172. doi:10.1007/s11199-006-9070-7

[bibr8-0361684317743019] *Aubrey J. S. (2007). The impact of sexually objectifying media exposure on negative body emotions and sexual self-perceptions: Investigating the mediating role of body self-consciousness. Mass Communication and Society, 10, 1–23. doi:10.1080/15205430709337002

[bibr9-0361684317743019] *Aubrey J. S. (2010). Looking good versus feeling good: An investigation of media frames of health advice and their effects on women’s body-related self-perceptions. Sex Roles, 63, 50–63. doi:10.1007/s11199-010-9768-4

[bibr10-0361684317743019] Aubrey J. S. Frisby C. M. (2011). Sexual objectification in music videos: A content analysis comparing gender and genre. Mass Communication and Society, 14, 475–501. doi:10.1080/15205436.2010.513468

[bibr11-0361684317743019] *Aubrey J. S. Gerding A. (2015). The cognitive tax of self-objectification. Journal of Media Psychology, 27, 22–32. doi:10.1027/1864-1105/a000128

[bibr12-0361684317743019] *Aubrey J. S. Henson J. R. Hopper K. M. Smith S. E. (2009). A picture is worth twenty words (about the self): Testing the priming influence of visual sexual objectification on women’s self-objectification. Communication Research Reports, 26, 271–284. doi:10.1080/08824090903293551

[bibr13-0361684317743019] *Aubrey J. S. Taylor L. D. (2005). Examining longitudinal relations between exposure to lad-genre media and undergraduates’ body self-consciousness. Paper presented at the annual conference of the International Communication Association (ICA), May 26–30, New York, NY.

[bibr14-0361684317743019] Aubrey J. S. Taylor L. D. (2009). The role of lad magazines in priming men’s chronic and temporary appearance-related schemata: An investigation of longitudinal and experimental findings. Human Communication Research, 35, 28–58. doi:10.1111/j.1468-2958.2008.01337.x

[bibr15-0361684317743019] Barlett C. P. Vowels C. L. Saucier D. A. (2008). Meta-analyses of the effects of media images on men’s body-image concerns. Journal of Social and Clinical Psychology, 27, 279–310. doi:10.1521/jscp.2008.27.3.279

[bibr16-0361684317743019] *Barzoki M. H. Mohtasham L. Shahidi M. Tavakol M. (2016). Self-objectification and self-sexualization behavior within consumer culture. Applied Research in Quality of Life, 11, 153–162. doi:10.1007/s11482-016-9468-5

[bibr17-0361684317743019] Bilandzic H. Rössler P. (2004). Life according to television. Implications of genre-specific cultivation effects: The gratification/cultivation model. Communications, 29, 295–326. doi:10.1515/comm.2004.020

[bibr18-0361684317743019] Borenstein M. Hedges L. V. Higgins J. P. Rothstein H. R. (2009). Introduction to meta-analysis. Statistics in practice. Chichester, England: John Wiley.

[bibr19-0361684317743019] Burgess M. C. Stermer S. P. Burgess S. R. (2007). Sex, lies, and video games: The portrayal of male and female characters on video game covers. Sex Roles, 57, 419–433. doi:10.1007/s11199-007-9250-0

[bibr20-0361684317743019] Calogero R. M. (2011). Operationalizing self-objectification: Assessment and related methodological issues In Calogero R. M. Tantleff-Dunn S. Thompson J. K. (Eds.), Self-objectification in women: Causes, consequences, and counteractions (pp. 23–50). Washington, DC: American Psychological Association.

[bibr21-0361684317743019] Calogero R. M. Herbozo S. Thompson J. K. (2009). Complimentary weightism: The potential costs of appearance-related commentary for women’s self-objectification. Psychology of Women Quarterly, 33, 120–132. doi:10.1111/j.1471-6402.2008.01479.x

[bibr22-0361684317743019] Calogero R. M. Tantleff-Dunn S. Thompson J. K. (2011). Future directions for research and practice In Calogero R. M. Tantleff-Dunn S. Thompson J. K. (Eds.), Self-objectification in women: Causes, consequences, and counteractions (pp. 217–237). Washington, DC: American Psychological Association.

[bibr23-0361684317743019] Capodilupo C. M. Kim S. (2015). Gender and race matter: The importance of considering intersections in Black women’s body image. Journal of Counseling Psychology, 61, 37–49. doi:10.1037/a0034597 10.1037/a0034597 24188651

[bibr24-0361684317743019] Cheung M. W. L. (2014). Modeling dependent effect sizes with three-level meta-analyses: A structural equation modeling approach. Psychological Methods, 19, 211–229. doi:10.1037/a0032968 23834422 10.1037/a0032968

[bibr25-0361684317743019] Choma B. L. Visser B. A. Pozzebon J. A. Bogaert A. F. Busseri M. A. Sadava S. W. (2010). Self-objectification, self-esteem, and gender: Testing a moderated mediation model. Sex Roles, 63, 645–656. doi:10.1007/s11199-010-9829-8

[bibr26-0361684317743019] Collins R. L. (2011). Content analysis of gender roles in media: Where are we now and where should we go? Sex Roles, 64, 290–298. doi:10.1007/s11199-010-9929-5

[bibr27-0361684317743019] *Dakanalis A. Di Mattei V. E. Bagliacca E. P. Prunas A. Sarno L. Riva G. Zanetti M. A. (2012). Disordered eating behaviors among Italian men: Objectifying media and sexual orientation differences. Eating Disorders, 20, 356–367. doi:10.1080/10640266.2012.715514 22985233 10.1080/10640266.2012.715514

[bibr28-0361684317743019] *Daniels E. A. (2009). Sex objects, athletes, and sexy athletes: How media representations of women athletes can impact adolescent girls and college women. Journal of Adolescent Research, 24, 399–422. doi:10.1177/0743558409336748

[bibr29-0361684317743019] de Guzman N. S. Nishina A. (2014). A longitudinal study of body dissatisfaction and pubertal timing in an ethnically diverse adolescent sample. Body Image, 11, 68–71. doi:10.1016/j.bodyim.2013.11.001 24331829 10.1016/j.bodyim.2013.11.001

[bibr30-0361684317743019] de Vreese C. H. Neijens P. (2016). Measuring media exposure in a changing communications environment. Communication Methods and Measures, 10, 69–80. doi:10.1080/19312458.2016.1150441

[bibr31-0361684317743019] *de Vries D. A. Peter J. (2013). Women on display: The effect of portraying the self online on women’s self-objectification. Computers in Human Behavior, 29, 1483–1489. doi:10.1016/j.chb.2013.01.015

[bibr32-0361684317743019] *Doornwaard S. M. Bickham D. S. Rich M. Vanwesenbeeck I. van den Eijnden R. J. J. M. ter Bogt T. F. M. (2014). Sex-related online behaviors and adolescents’ body and sexual self-perceptions. Pediatrics, 134, 1103–1110. doi:10.1542/peds.2014-0592 25404728 10.1542/peds.2014-0592

[bibr33-0361684317743019] Egger M. Smith G. D. Schneider M. Minder C. (1997). Bias in meta-analysis detected by a simple, graphical test. British Medical Journal, 315, 629–634. doi:10.1136/bmj.315.7109.629 9310563 10.1136/bmj.315.7109.629 PMC2127453

[bibr35-0361684317743019] Eveland W. P. (2003). A “mix of attributes” approach to the study of media effects and new communication technologies. Journal of Communication, 53, 395–410. doi:10.1093/joc/53.3.395

[bibr36-0361684317743019] *Fardouly J. Diedrichs P. C. Vartanian L. R. Halliwell E. (2015). The mediating role of appearance comparisons in the relationship between media usage and self-objectification in young women. Psychology of Women Quarterly, 39, 447–457. doi:10.1177/0361684315581841

[bibr37-0361684317743019] Field A. (2015). Dread returns to mega-silly one. Health Psychology Review, 9, 15–20. doi:10.1080/17437199.2013.879198 25793485 10.1080/17437199.2013.879198

[bibr38-0361684317743019] *Ford T. E. Woodzicka J. A. Petit W. E. Richardson K. Lappi S. K. (2015). Sexist humor as a trigger of state self-objectification in women. Humor, 28, 253–269. doi:10.1515/humor-2015-0018

[bibr39-0361684317743019] Fortenberry J. D. (2013). Sexual development in adolescents In Bromberg D. S. O’Donohue W. T. (Eds.), Handbook of child and adolescent sexuality. Developmental and forensic psychology (pp. 171–192). Amsterdam, The Netherlands: Elsevier.

[bibr40-0361684317743019] *Fox J. Bailenson J. N. Tricase L. (2013). The embodiment of sexualized virtual selves: The Proteus effect and experiences of self-objectification via avatars. Computers in Human Behavior, 29, 930–938. doi:10.1016/j.chb.2012.12.027

[bibr41-0361684317743019] *Fox J. Ralston R. A. Cooper C. K. Jones K. A. (2015). Sexualized avatars lead to women’s self-objectification and acceptance of rape myths. Psychology of Women Quarterly, 39, 349–362. doi:10.1177/0361684314553578

[bibr42-0361684317743019] *Fox J. Rooney M. C. (2015). The dark triad and trait self-objectification as predictors of men’s use and self-presentation behaviors on social networking sites. Personality and Individual Differences, 76, 161–165. doi:10.1016/j.paid.2014.12.017

[bibr43-0361684317743019] Fredrickson B. L. Hendler L. M. Nilsen S. O’Barr J. F. (2011). Bringing back the body: A retrospective on the development of objectification theory. Psychology of Women Quarterly, 35, 689–696. doi:10.1177/0361684311426690

[bibr44-0361684317743019] Fredrickson B. L. Roberts T.-A. (1997). Objectification theory: Toward understanding women’s lived experiences and mental health risks. Psychology of Women Quarterly, 21, 173–206. doi:10.1111/j.1471-6402.1997.tb00108.x

[bibr45-0361684317743019] Fredrickson B. L. Roberts T.-A. Noll S. M. Quinn D. M. Twenge J. M. (1998). That swimsuit becomes you: Sex differences in self-objectification, restrained eating, and math performance. Journal of Personality and Social Psychology, 75, 269–284. doi:10.1037/0022-3514.75.1.269 9686464 10.1037//0022-3514.75.1.269

[bibr46-0361684317743019] Galdi S. Maass A. Cadinu M. (2014). Objectifying media: Their effect on gender role norms and sexual harassment of women. Psychology of Women Quarterly, 38, 398–413. doi:10.1177/0361684313515185

[bibr47-0361684317743019] Gerbner G. (1998). Cultivation analysis: An overview. Mass Communication and Society, 1, 175–194. doi:10.1080/15205436.1998.9677855

[bibr48-0361684317743019] Gill R. (2009). Beyond the “sexualization of culture” thesis: An intersectional analysis of “sixpacks,” “midriffs” and “hot lesbians” in advertising. Sexualities, 12, 137–160. doi:10.1177/1363460708100916

[bibr49-0361684317743019] Grabe S. Hyde J. S. (2006). Ethnicity and body dissatisfaction among women in the United States: A meta-analysis. Psychological Bulletin, 132, 622–640. doi:10.1037/0033-2909.132.4.622 16822170 10.1037/0033-2909.132.4.622

[bibr50-0361684317743019] *Grabe S. Hyde J. S. (2009). Body objectification, MTV, and psychological outcomes among female adolescents. Journal of Applied Social Psychology, 39, 2840–2858. doi:10.1111/j.1559-1816.2009.00552.x

[bibr51-0361684317743019] Grabe S. Ward L. M. Hyde J. S. (2008). The role of the media in body image concerns among women: A meta-analysis of experimental and correlational studies. Psychological Bulletin, 134, 460–476. doi:10.1037/0033-2909.134.3.460 18444705 10.1037/0033-2909.134.3.460

[bibr52-0361684317743019] *Grey M. J. Horgan T. G. Long T. A. Herzog N. K. Lindemulder J. R. (2016). Contrasting objectification and competence. Journal of Media Psychology, 28, 88–93. doi:10.1027/1864-1105/a000159

[bibr53-0361684317743019] Groesz L. M. Levine M. P. Murnen S. K. (2002). The effect of experimental presentation of thin media images on body satisfaction: A meta-analytic review. International Journal of Eating Disorders, 31, 1–16. doi:10.1002/eat.10005 11835293 10.1002/eat.10005

[bibr54-0361684317743019] Guo S. (2016). A meta-analysis of the predictors of cyberbullying perpetration and victimization. Psychology in Schools, 53, 432–453. doi:10.1002/pits.21914

[bibr55-0361684317743019] Hall P. C. West J. H. McIntyre E. (2012). Female self-sexualization in MySpace.com personal profile photographs. Sexuality & Culture, 16, 1–16. doi:10.1007/s12119-011-9095-0

[bibr56-0361684317743019] *Halliwell E. Malson H. Tischner I. (2011). Are contemporary media images which seem to display women as sexually empowered actually harmful to women? Psychology of Women Quarterly, 35, 38–45. doi:10.1177/0361684310385217

[bibr57-0361684317743019] *Harper B. Tiggemann M. (2008). The effect of thin ideal media images on women’s self-objectification, mood, and body image. Sex Roles, 58, 649–657. doi:10.1007/s11199-007-9379-x

[bibr58-0361684317743019] *Harrison K. Fredrickson B. L. (2003). Women’s sports media, self-objectification, and mental health in black and white adolescent females. Journal of Communication, 1, 216–232. doi:10.1017/S0140525X00047257

[bibr59-0361684317743019] Harrison K. Hefner V. (2014). Virtually perfect: Image retouching and adolescent body image. Media Psychology, 17, 134–153. doi:10.1080/15213269.2013.770354

[bibr60-0361684317743019] Hatton E. Trautner M. N. (2011). Equal opportunity objectification? The sexualization of men and women on the cover of Rolling Stone. Sexuality and Culture, 15, 256–278. doi:10.1007/s12119-011-9093-2

[bibr61-0361684317743019] Hausenblas H. A. Campbell A. Menzel J. E. Doughty J. Levine M. Thompson J. K. (2013). Media effects of experimental presentation of the ideal physique on eating disorder symptoms: A meta-analysis of laboratory studies. Clinical Psychology Review, 33, 168–181. doi:10.1016/j.cpr.2012.10.011 23232051 10.1016/j.cpr.2012.10.011

[bibr62-0361684317743019] Hedges L. V. Vevea J. L. (1998). Fixed- and random-effects models in meta-analysis. Psychological Methods, 3, 486–504. doi:10.1037/1082-989X.3.4.486

[bibr63-0361684317743019] Higgins J. P. T. Green S (2011). Cochrane handbook for systematic reviews of interventions (Version 5.1.0). Retrieved from www.cochrane-handbook.org

[bibr64-0361684317743019] Higgins J. P. T. Thompson S. G. (2002). Quantifying heterogeneity in a meta-analysis. Statistics in Medicine, 21, 1539–1558. doi:10.1002/sim.1186 12111919 10.1002/sim.1186

[bibr65-0361684317743019] *Hofschire L. (2003). The media’s role in enhancing self-objectification and eating disorders. Paper presented at the annual conference of the International Communication Association (ICA), May 23–27, San Diego, CA.

[bibr66-0361684317743019] Holland E. Haslam N. (2016). Cute little things. Psychology of Women Quarterly, 40, 108–119. doi:10.1177/0361684315602887

[bibr67-0361684317743019] Holland G. Tiggemann M. (2016). A systematic review of the impact of the use of social networking sites on body image and disordered eating outcomes. Body Image, 17, 100–110. doi:10.1016/j.bodyim.2016.02.008 26995158 10.1016/j.bodyim.2016.02.008

[bibr68-0361684317743019] Holmstrom A. J. (2004). The effects of the media on body image: A meta-analysis. Journal of Broadcasting & Electronic Media, 48, 196–217. doi:10.1207/s15506878jobem4802_3

[bibr69-0361684317743019] *Hopper K. M. Aubrey J. S. (2013). Examining the impact of celebrity gossip magazine coverage of pregnant celebrities on pregnant women’s self-objectification. Communication Research, 40, 767–788. doi:10.1177/0093650211422062

[bibr70-0361684317743019] *Hopper K. M. Aubrey J. S. (2016). Bodies after babies: The impact of depictions of recently post-partum celebrities on non-pregnant women’s body image. Sex Roles, 74, 24–34. doi:10.1007/s11199-015-0561-2

[bibr71-0361684317743019] Huedo-Medina T. B. Sánchez-Meca J. Marín-Martínez F. Botella J. (2006). Assessing heterogeneity in meta-analysis: Q statistic or I2 index? Psychological Methods, 11, 193–206. doi:10.1037/1082-989X.11.2.193 16784338 10.1037/1082-989X.11.2.193

[bibr72-0361684317743019] Hunter J. E. Schmidt F. L. (2004). Methods of meta-analysis: Correction error and bias in research findings. Thousand Oaks, CA: Sage.

[bibr73-0361684317743019] Jongenelis M. I. Byrne S. M. Pettigrew S. (2014). Self-objectification, body image disturbance, and eating disorder symptoms in young Australian children. Body Image, 11, 290–302. doi:10.1016/j.bodyim.2014.04.002 24958665 10.1016/j.bodyim.2014.04.002

[bibr74-0361684317743019] *Kalodner C. R. (1997). Media influences on male and female non-eating-disordered college students: A significant issue. Eating Disorders, 5, 47–57. doi:10.1080/10640269708249203

[bibr75-0361684317743019] Kapidzic S. Herring S. C. (2015). Race, gender, and self-presentation in teen profile photographs. New Media & Society, 17, 958–976. doi:10.1177/1461444813520301

[bibr76-0361684317743019] *Karsay K. Matthes J. (2015). Sexualizing pop music videos, self-objectification, and selective exposure: A moderated mediation model. Paper presented at the annual conference of the Association for Education in Journalism and Mass Communication (AEJMC), August 6–9, San Francisco, CA.

[bibr77-0361684317743019] Karsay K. Matthes J. (2016). Sexually objectifying pop music videos, young women’s self objectification, and selective exposure: A moderated mediation model. Communication Research, 1–22. doi:10.1177/0093650216661434

[bibr78-0361684317743019] *Kim S. Y. Seo Y. S. Baek K. Y. (2015). Face consciousness among South Korean women: A culture-specific extension of objectification theory. Journal of Counseling Psychology, 61, 24–36. doi:10.1037/a0034433 10.1037/a0034433 24040778

[bibr79-0361684317743019] Klaassen J. E. Peter J. (2015). Gender (in)equality in internet pornography: A content analysis of popular pornographic internet videos. The Journal of Sex Research, 52, 721–735. doi:10.1080/00224499.2014.976781 25420868 10.1080/00224499.2014.976781

[bibr80-0361684317743019] Konstantopoulos S. (2011). Fixed effects and variance components estimation in three-level meta-analysis. Research Synthesis Methods, 2, 61–76. doi:10.1002/jrsm.35 26061600 10.1002/jrsm.35

[bibr81-0361684317743019] Kozee H. B. Tylka T. L. Augustus-Horvath C. L. Denchik A. (2007). Development and psychometric evaluation of the interpersonal sexual objectification scale. Psychology of Women Quarterly, 31, 176–189. doi:10.1111/j.1471-6402.2007.00351.x

[bibr82-0361684317743019] Krippendorff K. (2004). Content analysis: An introduction to its methodology. Thousand Oaks, CA: Sage.

[bibr83-0361684317743019] Levine M. P. Murnen S. K. (2009). “Everybody knows that mass media are/are not [pick one] a cause of eating disorders”: A critical review if evidence for a causal link between media, negative body image, and disordered eating in females. Journal of Social and Clinical Psychology, 28, 9–42. doi:10.1521/jscp.2009.28.1.9

[bibr84-0361684317743019] Lindberg S. M. Hyde J. S. McKinley N. M. (2006). A measure of objectified body consciousness for preadolescent and adolescent youth. Psychology of Women Quarterly, 30, 65–76. doi:10.1111/j.1471-6402.2006.00263.x

[bibr85-0361684317743019] Lipsey M. W. Wilson D. B. (2001). Practical meta-analysis. Applied social research methods series (Vol. 49). Thousand Oaks, CA: Sage.

[bibr86-0361684317743019] López-Guimerà G. Levine M. P. Sánchez-carracedo D. Fauquet J. (2010). Influence of mass media on body image and eating disordered attitudes and behaviors in females: A review of effects and processes. Media Psychology, 13, 387–416. doi:10.1080/15213269.2010.525737

[bibr87-0361684317743019] Lynch T. Tompkins J. E. van Driel I. I. Fritz N. (2016). Sexy, strong, and secondary: A content analysis of female characters in video games across 31 years. Journal of Communication, 66, 564–584. doi:10.1111/jcom.12237

[bibr88-0361684317743019] *Manago A. M. Ward L. M. Lemm K. M. Reed L. Seabrook R. (2015). Facebook involvement, objectified body consciousness, body shame, and sexual assertiveness in college women and men. Sex Roles, 72, 1–14. doi:10.1007/s11199-014-0441 -1

[bibr89-0361684317743019] McKinley N. M. Hyde J. S. (1996). The objectified body consciousness scale: Development and validation. Psychology of Women Quarterly, 20, 181–215. doi:1111/j.1471-6402.1996.tb00467.x

[bibr90-0361684317743019] *Meier E. P. Gray J. (2014). Facebook photo activity associated with body image disturbance in adolescent girls. Cyberpsychology, Behavior and Social Networking, 17, 199–206. doi:10.1089/cyber.2013.0305 10.1089/cyber.2013.0305 24237288

[bibr91-0361684317743019] *Melioli T. Rodgers R. F. Rodrigues M. Chabrol H. (2015). The role of body image in the relationship between internet use and bulimic symptoms: Three theoretical frameworks. Cyberpsychology, Behavior, and Social Networking, 18, 682–687. doi:10.1089/cyber.2015.0154 10.1089/cyber.2015.0154 26378881

[bibr93-0361684317743019] *Michaels M. S. Parent M. C. Moradi B. (2013). Does exposure to muscularity-idealizing images have self-objectification consequences for heterosexual and sexual minority men? Psychology of Men & Masculinity, 14, 175–183. doi:10.1037/a0027259

[bibr94-0361684317743019] Miller L. C. Murphy R. Buss A. H. (1981). Consciousness of body: Private and public. Journal of Personality and Social Psychology, 41, 397–406. doi:10.1037/0022-3514.41.2.397

[bibr95-0361684317743019] *Miller M. A. (2007). Effects of highly sexualized images of women in visual media on adolescent females’ objectified body consciousness and feminine ideology (Unpublished doctoral dissertation). Walden University, Minneapolis, MN.

[bibr96-0361684317743019] Moradi B. (2010). Addressing gender and cultural diversity in body image: Objectification theory as a framework for integrating theories and grounding research. Sex Roles, 63, 138–148. doi:10.1007/s11199-010-9824-0

[bibr97-0361684317743019] Moradi B. (2011). Objectification theory: Areas of promise and refinement. The Counseling Psychologist, 39, 153–163. doi:10.1177/0011000010384279

[bibr98-0361684317743019] Moradi B. Huang Y.-P. (2008). Objectification theory and psychology of women: A decade of advances and future directions. Psychology of Women Quarterly, 32, 377–398. doi:10.1111/j.1471-6402.2008.00452.x

[bibr99-0361684317743019] *Morry M. M. Staska S. L. (2001). Magazine exposure: Internalization, self-objectification, eating attitudes, and body satisfaction in male and female university students. Canadian Journal of Behavioural Science/Revue Canadienne Des Sciences Du Comportement, 33, 269–279. doi:10.1037/h0087148

[bibr100-0361684317743019] *Nabi R. L. (2009). Cosmetic surgery makeover programs and intentions to undergo cosmetic enhancements: A consideration of three models of media effects. Human Communication Research, 35, 1–27. doi:10.1111/j.1468-2958.2008.01336.x

[bibr101-0361684317743019] Noll S. M. Fredrickson B. L. (1998). A mediational model linking self-objectification, body shame, and disordered eating. Psychology of Women Quarterly, 22, 623–636. doi:10.1111/j.1471-6402.1998.tb00181.x

[bibr102-0361684317743019] *Nowatzki J. Morry M. M. (2009). Women’s intentions regarding, and acceptance of, self-sexualizing behavior. Psychology of Women Quarterly, 33, 95–107. doi:10.1111/j.1471-6402.2008.01477.x

[bibr92-0361684317743019] O’Keefe D. J. (2017). Misunderstandings of effect sizes in message effects research. Communication Methods and Measures, 11, 210–219. doi:10.1080/19312458.2017.1343812

[bibr103-0361684317743019] Overstreet N. M. Quinn D. M. Agocha V. B. (2010). Beyond thinness: The influence of a curvaceous body ideal on body dissatisfaction in black and white women. Sex Roles, 63, 91–103. doi:10.1007/s11199-010-9792-4

[bibr104-0361684317743019] Pearce L. J. Field A. P. (2016). The impact of “scary” TV and film on children’s internalizing emotions: A meta-analysis. Human Communication Research, 42, 98–121. doi:10.1111/hcre.12069

[bibr105-0361684317743019] Perloff R. M. (2014). Social media effects on young women’s body image concerns: Theoretical perspectives and an agenda for research. Sex Roles, 71, 363–377. doi:10.1007/s11199-014-0384-6

[bibr106-0361684317743019] Peterson R. A. Brown S. P. (2005). On the use of beta coefficients in meta-analysis. Journal of Applied Psychology, 90, 175–181. doi:10.1037/0021-9010.90.1.175 15641898 10.1037/0021-9010.90.1.175

[bibr107-0361684317743019] Pigott T. (2012). Advances in meta-analysis. New York, NY: Springer.

[bibr108-0361684317743019] Piran N. (2015). New possibilities in the prevention of eating disorders: The introduction of positive body image measures. Body Image, 14, 146–157. doi:10.1016/j.bodyim.2015.03.008 25886711 10.1016/j.bodyim.2015.03.008

[bibr109-0361684317743019] Piran N. (2016). Embodied possibilities and disruptions: The emergence of the experience of embodiment construct from qualitative studies with girls and women. Body Image, 18, 43–60. doi:10.1016/j.bodyim.2016.04.007 27236476 10.1016/j.bodyim.2016.04.007

[bibr110-0361684317743019] *Prichard I. Tiggemann M. (2012). The effect of simultaneous exercise and exposure to thin-ideal music videos on women’s state self-objectification, mood and body satisfaction. Sex Roles, 67, 201–210. doi:10.1007/s11199-012-0167-x

[bibr111-0361684317743019] Ricciardelli R. Clow K. A. White P. (2010). Investigating hegemonic masculinity: Portrayals of masculinity in men’s lifestyle magazines. Sex Roles, 63, 64–78. doi:10.1007/s11199-010-9764-8

[bibr112-0361684317743019] Rohlinger D. A. (2002). Eroticizing men: Cultural influences on advertising and male objectification. Sex Roles, 46, 61–74. doi:10.1023/A:1016575909173

[bibr113-0361684317743019] Rosenthal R. (1979). The file drawer problem and tolerance for null results. Psychological Bulletin, 68, 638–641. doi:10.1037/0033-2909.86.3.638

[bibr114-0361684317743019] Rosenthal R. DiMatteo M. R. (2001). Meta-analysis. Recent developments in quantitative methods for literature reviews. Annual Review of Psychology, 52, 59–82. doi:10.1146/annurev.psych.52.1.59 10.1146/annurev.psych.52.1.59 11148299

[bibr115-0361684317743019] *Slater A. Tiggemann M. (2015). Media exposure, extracurricular activities, and appearance-related comments as predictors of female adolescents’ self-objectification. Psychology of Women Quarterly, 39, 375–389. doi:10.1177/0361684314554606

[bibr116-0361684317743019] Slater A. Tiggemann M. (2016). Little girls in a grown up world: Exposure to sexualized media, internalization of sexualization messages, and body image in 6-9 year-old girls. Body Image, 18, 19–22. doi:10.1016/j.bodyim.2016.04.004 27236473 10.1016/j.bodyim.2016.04.004

[bibr117-0361684317743019] Stankiewicz J. M. Rosselli F. (2008). Women as sex objects and victims in print advertisements. Sex Roles, 58, 579–859. doi:10.1007/s11199-007-9359 -1

[bibr118-0361684317743019] Swami V. Frederick D. A. Aavik T. Alcalay L. Allik J. Anderson D.…Zivcic-Becirevic I. (2010). The attractive female body weight and female body dissatisfaction in 26 countries across 10 world regions: Results of the International Body Project I. Personality and Social Psychology Bulletin, 36, 309–325. doi:10.1177/0146167209359702 20179313 10.1177/0146167209359702

[bibr119-0361684317743019] Swim J. K. Hyers L. L. Cohen L. L. Ferguson M. J. (2001). Everyday sexism: Evidence for its incidence, nature, and psychological impact from three daily diary studies. Journal of Social Issues, 57, 31–53. doi:10.1111/0022-4537.00200

[bibr120-0361684317743019] Thompson J. K. van den Berg P. Roehrig M. Guarda A. S. Heinberg L. J. (2004). The sociocultural attitudes towards appearance scale-3 (SATAQ-3): Development and validation. International Journal of Eating Disorders, 35, 293–304. doi:10.1002/eat.10257 15048945 10.1002/eat.10257

[bibr121-0361684317743019] *Tiggemann M. Slater A. (2013). NetGirls: The internet, Facebook, and body image concern in adolescent girls. International Journal of Eating Disorders, 46, 630–633. doi:10.1002/eat.22141 23712456 10.1002/eat.22141

[bibr122-0361684317743019] *Tiggemann M. Slater A. (2014). NetTweens: The internet and body image concerns in preteenage girls. The Journal of Early Adolescence, 34, 606–620. doi:10.1177/0272431613501083

[bibr123-0361684317743019] *Tiggemann M. Slater A. (2015). The role of self-objectification in the mental health of early adolescent girls: Predictors and consequences. Journal of Pediatric Psychology, 40, 704–711. doi:10.1093/jpepsy/jsv021 25810536 10.1093/jpepsy/jsv021

[bibr124-0361684317743019] Tolman D. L. Porche M. V. (2000). The adolescent femininity ideology scale. Development and validation of a new measure for girls. Psychology of Women Quarterly, 24, 365–376. doi:10.1111/j.1471-6402.2000.tb00219.x

[bibr125-0361684317743019] *Tylka T. L. (2015). No harm in looking, right? Men’s pornography consumption, body image, and well-being. Psychology of Men & Masculinity, 16, 97–107. doi:10.1037/a0035774

[bibr127-0361684317743019] Valkenburg P. M. Peter J (2013). Five challenges for the future of media-effects research. International Journal of Communication, 7, 197–215. doi:932–8036/2013FEA0002

[bibr128-0361684317743019] van Oosten J. M. F. Peter J. Boot I. (2015). Women’s critical responses to sexually explicit material: The role of hyperfemininity and processing style. The Journal of Sex Research, 52, 306–316. doi:10.1080/00224499.2013.858305 24511896 10.1080/00224499.2013.858305

[bibr129-0361684317743019] *Vandenbosch L. Driesmans K. Trekels J. Eggermont S. (2015). The effect of playing with video game avatars on self-objectification in adolescent boys and girls. Paper presented at the annual conference of the International Communication Association (ICA), May 21–25, San Juan, Puerto Rico.

[bibr130-0361684317743019] *Vandenbosch L. Eggermont S. (2012). Understanding sexual objectification: A comprehensive approach toward media exposure and girls’ internalization of beauty ideals, self-objectification, and body surveillance. Journal of Communication, 62, 869–887. doi:10.1111/j.1460-2466.2012.01667.x

[bibr131-0361684317743019] *Vandenbosch L. Eggermont S. (2013). Sexualization of adolescent boys: Media exposure and boys’ internalization of appearance ideals, self-objectification, and body surveillance. Men and Masculinities, 16, 283–306. doi:10.1177/1097184X13477866

[bibr132-0361684317743019] *Vandenbosch L. Eggermont S. (2014). The role of television in adolescents’ sexual attitudes: Exploring the explanatory value of the three-step self-objectification process. Poetics, 45, 19–35. doi:10.1016/j.poetic.2014.06.002 10.1007/s10508-014-0292-4 24789048

[bibr133-0361684317743019] *Vandenbosch L. Eggermont S. (2015a). The interrelated roles of mass media and social media in adolescents’ development of an objectified self-concept: A longitudinal study. Communication Research, 43, 1116–1140. doi:10.1177/0093650215600488

[bibr134-0361684317743019] *Vandenbosch L. Eggermont S. (2015b). The role of mass media in adolescents’ sexual behaviors: Exploring the explanatory value of the three-step self-objectification process. Archives of Sexual Behavior, 44, 729–742. doi:10.1007/s10508-014-0292-4 24789048 10.1007/s10508-014-0292-4

[bibr135-0361684317743019] *Vandenbosch L. Muise A. Eggermont S. Impett E. A. (2015). Sexualizing reality television: Associations with trait and state self-objectification. Body Image, 13, 62–66. doi:10.1016/j.bodyim.2015.01.003 25687937 10.1016/j.bodyim.2015.01.003

[bibr136-0361684317743019] Vandenbosch L. Vervloessem D. Eggermont S (2013). “I might get your heart racing in my skin-tight jeans”: Sexualization on music entertainment television. Communication Studies, 64, 178–194. doi:10.1080/10510974.2012.755640

[bibr137-0361684317743019] Veldhuis J. Konijn E. A. Seidell J. C. (2014). Counteracting media’s thin-body ideal for adolescent girls: Informing is more effective than warning. Media Psychology, 17, 154–184. doi:10.1080/15213269.2013.788327

[bibr138-0361684317743019] Viechtbauer W. (2010). Conducting meta-analyses in R with the metaphor package. Journal of Statistical Software, 36, 1–48. doi:10.18637/jss.v036.i03

[bibr139-0361684317743019] Viechtbauer W. (2015). Package metafor. Retrieved from https://cran.r-project.org/web/packages/metafor/metafor.pdf

[bibr140-0361684317743019] *Volgman M. E. (2014). More than music to my ears: Music lyrics and self-objectification (Unpublished doctoral dissertation). Fielding Graduate University, Santa Barbara, CA.

[bibr141-0361684317743019] Want S. C. (2009). Meta-analytic moderators of experimental exposure to media portrayals of women on female appearance satisfaction: Social comparisons as automatic processes. Body Image, 6, 257–269. doi:10.1016/j.bodyim.2009.07.008 19716779 10.1016/j.bodyim.2009.07.008

[bibr142-0361684317743019] Ward L. M. (2016). Media and sexualization: State of empirical research, 1995–2015. The Journal of Sex Research, 53, 1–18. doi:10.1080/00224499.2016.1142496 26979592 10.1080/00224499.2016.1142496

[bibr143-0361684317743019] *Ward L. M. Seabrook R. C. Manago A. Reed L. (2015). Contributions of diverse media to self-sexualization among undergraduate women and men. Sex Roles, 74, 12–23. doi:10.1007/s11199-015-0548-z

[bibr144-0361684317743019] Weibel D. Wissmath B. Mast F. W. (2011). Influence of mental imagery on spatial presence and enjoyment assessed in different types of media. CyberPsychology, Behavior & Social Networking, 14, 607–612. doi:10.1089/cyber.2010.0287 10.1089/cyber.2010.0287 21352082

[bibr145-0361684317743019] Wirth W. Hartmann T. Böcking S. Vorderer P. Klimmt C. Schramm H.…Jäncke P. (2007). A process model of the formation of spatial presence experiences. Media Psychology, 9, 493–525. doi:10.1080/15213260701283079

[bibr146-0361684317743019] Zurbriggen E. L. (2013). Objectification, self-objectification, and societal change. Journal of Social and Political Psychology, 1, 188–215. doi:10.5964/jspp.v1i1.94

[bibr147-0361684317743019] *Zurbriggen E. L. Ramsey L. R. Jaworski B. K. (2011). Self- and partner-objectification in romantic relationships: Associations with media consumption and relationship satisfaction. Sex Roles, 64, 449–462. doi:10.1007/s11199-011-9933-4 21475650 10.1007/s11199-011-9933-4 PMC3062032

